# A Cold and Its Consequences

**Published:** 1872-10

**Authors:** 


### A COLD AND ITS CONSEQUENCES.

A PRESENT of a pig, ready for the roasting, was sent to a lady of wealth, the mother of several growing children; not wishing to place it on her own table, she ordered it to be "pickled," with the intention of distributing it to several poor and deserving families in the neighborhood.

On a warm spring day, she had been engaged in some occupation which heated the body somewhat above its natural condition; and just at that particular juncture, some unusual occurrence seemed to make it desirable that she should select a piece of the pickled pork for a particular, and even critical purpose, otherwise a servant could have readily attended to the matter. She handled several pieces, dipping her hands and above the wrists in and out of the "brine" for a minute or two. Within a very short time after, a pain darted across the upper chest, of such violence as to cause a slight scream; a chill passed over the body, a paralysis of muscle and mind and brain followed, succeeded by an attack of inflammatory rheumatism, which confined her to her bed for six months, with its intolerable pains; her feet became swollen and bursted; for many weeks she could not walk without crutches, and at the end of twenty years, was unable to bear the slightest draught of air, as such a thing always resulted in violent neuralgias, extending all over the body. In this case two errors were committed; first, in putting the hands in any liquid, while in the slightest perspiration; second, in the imprudence of putting the hands in salt water when thus overheated, because it is much colder than common water, although exposed to the same temperature.

